# Population Growth Rates of Reef Sharks with and without Fishing on the Great Barrier Reef: Robust Estimation with Multiple Models

**DOI:** 10.1371/journal.pone.0025028

**Published:** 2011-09-23

**Authors:** Mizue Hisano, Sean R. Connolly, William D. Robbins

**Affiliations:** 1 School of Marine and Tropical Biology, James Cook University, Townsville, Australia; 2 ARC Centre of Excellence for Coral Reef Studies, James Cook University, Townsville, Australia; University of Alberta, Canada

## Abstract

Overfishing of sharks is a global concern, with increasing numbers of species threatened by overfishing. For many sharks, both catch rates and underwater visual surveys have been criticized as indices of abundance. In this context, estimation of population trends using individual demographic rates provides an important alternative means of assessing population status. However, such estimates involve uncertainties that must be appropriately characterized to credibly and effectively inform conservation efforts and management. Incorporating uncertainties into population assessment is especially important when key demographic rates are obtained via indirect methods, as is often the case for mortality rates of marine organisms subject to fishing. Here, focusing on two reef shark species on the Great Barrier Reef, Australia, we estimated natural and total mortality rates using several indirect methods, and determined the population growth rates resulting from each. We used bootstrapping to quantify the uncertainty associated with each estimate, and to evaluate the extent of agreement between estimates. Multiple models produced highly concordant natural and total mortality rates, and associated population growth rates, once the uncertainties associated with the individual estimates were taken into account. Consensus estimates of natural and total population growth across multiple models support the hypothesis that these species are declining rapidly due to fishing, in contrast to conclusions previously drawn from catch rate trends. Moreover, quantitative projections of abundance differences on fished versus unfished reefs, based on the population growth rate estimates, are comparable to those found in previous studies using underwater visual surveys. These findings appear to justify management actions to substantially reduce the fishing mortality of reef sharks. They also highlight the potential utility of rigorously characterizing uncertainty, and applying multiple assessment methods, to obtain robust estimates of population trends in species threatened by overfishing.

## Introduction

There is mounting evidence of widespread, substantial, and ongoing declines in the abundance of shark populations worldwide, coincident with marked rises in global shark catches in the last half-century [1]–[3]. In some cases, these declines have been linked to resultant trophic cascades [1], [4]. Consequently, overfishing of sharks is now recognized as a major global conservation concern [5], with increasing numbers of shark species added to the International Union for the Conservation of Nature's list of threatened species [6]. However, our knowledge of the status of many shark populations is limited due to lack of, or ambiguous data [7].

**Figure 1:**
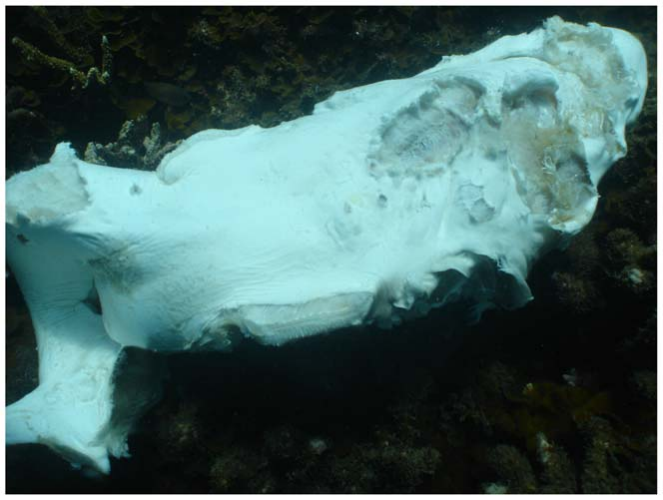
A finned reef shark in the Great Barrier Reef World Heritage Area. This carcass was one of several found illegally dumped at Wreck Beach on Great Keppel Island (Photo taken by R. Berkelmans on 17 November 2006).

On coral reefs, apex predators, including medium-sized reef sharks, can make up a large proportion of fish biomass in the absence of fishing [8], [9]. Food web models suggest that they also are strongly interacting: per capita, they have relatively strong effects on other species in the community [10]. However, evaluating population trends for reef shark species, like that of many sharks, is complicated by several factors that make trends in reported catch and catch rate data unreliable indicators of fishing mortality or abundance. Firstly, many countries with significant coral reefs do not have extensive and reliable reporting of total catches, or of fishing effort [11], both of which are required to obtain fisheries-based indices of abundance. Indeed, even where catch and effort data are available, there is often little information about covariates needed to standardize the catch-effort relationship, such as changes in gear types or targeting behavior of the fishery. Secondly, a large proportion of the global catch consists of illegal (and therefore unreported) shark finning: a recent estimate based on fin-trade data identified 75% of the global shark catch as illegal and unreported [12]. Reef sharks are a small, but acknowledged part of such catches [13], [14]. Such activity can even occur in intensively managed reef systems (Figure 1). Thirdly, sharks may be caught as bycatch in fisheries targeting other species; often these sharks are not reported at the species level [3], or are killed and discarded at sea, and not recorded as catch [7], [15]. Finally, robust inference of population trends from catch data requires lengthy time series, precluding timely use when decades of high-quality catch records are unavailable [16].

In part due to lack of availability of catch data, underwater visual surveys (UVS) are becoming an increasingly common method to assess the status of shallow-water shark populations. Worldwide, most of the key evidence for reef shark depletion comes from such data [9], [14], [17], [18]. However, for highly mobile fishes such as sharks, UVS has been criticized for potentially leading to severe biases in estimates of abundances [19]–[21], including comparisons of fished versus unfished locations [22]. For instance, if sharks are more unaccustomed to people in unfished areas (remote locations or no-entry protected areas), then they may be more likely to approach researchers, leading to over-estimates of the effects of fishing.

An alternative to inferring population status from catch rates or UVS is to parameterize a population model from estimates of growth rates, fecundity, maturity schedules, and mortality rates, to estimate the long-term population growth rate. In such analyses, age, maturity, and fecundity can be obtained directly from captured individuals. However, estimation of mortality rates is less straightforward. For many fishes, including sharks, sufficient mark-recapture data are rarely available, due to problems of the relative rarity of the animals and low rates of recapture (usually ∼5% or less for sharks: [23]). Consequently, for these species, mortality is frequently estimated from characteristics of the population as a whole, such as the age structure of the catch (catch-curve analysis: [24], [25]). Alternatively, indirect methods that infer mortality rate from putative relationships with other, easier-to-measure life history parameters may be used. These relationships may be empirical [26], [27], or derived from life history theory [28].

Given the lack of consensus in the literature about the reliability of catch rate trends and UVS for assessment of shark population status, an evaluation of the robustness and consistency of population growth rates derived from alternative methods of estimating mortality rate is needed. Inference based on multiple models is increasingly recognized as an important way to reduce the biases associated with the particular simplifying assumptions of individual models, and it is now widely applied in a variety of environmental policy contexts, from the management of threatened species [29], [30], to the estimation of climate sensitivity [31]. Therefore, in this study, we explore the use of multiple models, and rigorous characterization of uncertainty, to assess mortality and population growth rates of sharks, focusing on two species on the Great Barrier Reef (GBR), Australia: the grey reef shark *Carcharhinus amblyrhynchos* and the whitetip reef shark *Triaenodon obesus*. Previous estimates of these species' population growth rates, based on catch-curve analysis, suggested ongoing, rapid population declines [18]. This is qualitatively consistent with differences in visual abundance estimates on fished and unfished reefs [18], [32]. However, analysis of time series of catch rates has failed to find statistically significant population declines [33]. Here, we estimate natural and total (i.e., including fishing-induced) mortality rates using several indirect methods, and we determine the corresponding population growth rates implied by each of these mortality estimates. We use bootstrapping to comprehensively quantify the uncertainty associated with each of our estimates, and to correct for biases associated with the estimation process. We also use this characterization of uncertainty to critically evaluate the extent of agreement between indirect and catch curve-based methods for estimating population growth rate, and to produce “consensus” estimates of natural and total population growth rate. Finally, we combine our consensus estimates of natural and total population growth rates to estimate the rate of growth of abundance differences between fished and unfished populations, and we compare these projected abundance differences with previous estimates from UVS data on fished and unfished reefs.

## Methods

### Growth, Maturity, and Fecundity

The age-specific maturity and fecundity data for our two study species, and the catch data used in the catch curve analysis, have previously been described in detail [18]. However, we summarize these data and associated parameter estimation in the Supplementary Material (Text S1). For some of the methods described below, growth parameters are also required. Therefore, we also fitted the three-parameter von Bertalanffy growth function (VBGF) to size-at-age data:
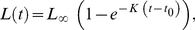
(1)


where *L*(*t*) is length at age *t*, *t* is age, and 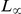, *K*, and *t*_0_ are estimated parameters indicating the mean asymptotic length, rate of growth towards the asymptotic length, and hypothetical age at size zero [34]. This model, fitted by ordinary least-squares, provided a good fit to our data (Text S1).

### Estimation of Total Mortality Rates (*Z*)

#### Catch curves (*Z_CC_*)

For this method, we used estimates based on a previous analysis of catch curves [18]. This approach typically entails a linear regression of log-transformed frequency against age, the slope of which is the total instantaneous mortality rate. In practice, the age that is most represented in the catch is assumed to represent the age at which individuals have fully recruited to the fishery, and the regression is confined to individuals at or above this age. For *C. amblyrhynchos*, however, there was strong evidence against a constant mortality rate [18], so a non-linear regression was used to estimate age-specific mortality. This approach is questionable because one possible cause of age-dependent mortality is higher susceptibility to fishing of younger, naive individuals. Therefore, we also estimate mortality rate for this species using the catch curve of *T. obesus* (*Z_CCT_*), whose population structure above age 5 is more consistent with a constant mortality rate after sharks have recruited to the fishery. Because *C. amblyrhynchos* is known to be considerably more aggressive towards bait than *T. obesus* [35] and because natural mortality estimates for these two species tended to be very similar (see Results), this latter approach is likely to be biased low as an estimate of total mortality rate for *C. amblyrhynchos*.

#### Beverton-Holt (*Z_BH_*)

This method relates the mean age of animals in the catch to total mortality rate. If recruitment rate is constant, and mortality rate is independent of age after recruitment, then total mortality rate can be estimated from the relationship:
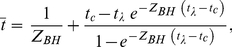
(2)


where 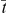 is the mean age (in years) in the catch, 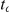 is the age of recruitment to the fishing gear, and 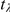
is longevity [36]. Equation 2 has no explicit solution, but can be solved numerically for *Z_BH_* given estimates of 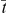 (9.2 years for *T. obesus*, and 4.3 years for *C. amblyrhynchos*, obtained from the catch data), 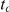 (5 for *T*. obesus; 0 for *C. amblyrhynchos*), and 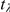 (25 for both species for the figures in the main text, but see below for a description of our sensitivity analysis for longevity).

#### Hoenig (*Z_HF_* and *Z_HC_*)

This method exploits an empirical relationship between observed longevity and mortality rate of animals. Specifically, for fish, cetaceans, and mollusks, the relationship between species-specific estimates of mortality rate, and the maximum observed age of those species, is well-described by a linear regression in log-log space:
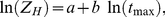
(3)


where *a* and *b* are fitted regression parameters, and *t*_max_ is maximum observed age in the catch (19 years for both species) [27]. For sharks, the regression parameters from the analysis of fish (*a* = 1.46, *b* = −1.01) are often used (hereafter *Z_HF_*). However, because demographic characteristics of sharks often resemble those of cetaceans more closely than teleost fishes [37], we also employ the regression parameters obtained for cetaceans (*a* = 0.941, *b* = −0.873: hereafter *Z_HC_*).

### Estimation of Natural Mortality Rates (*M*)

#### Pauly (*M_P_*)

Like *Z_HF_* and *Z_HC_*, this method relies on empirical relationships between species-specific estimates of mortality rate, and other characteristics of those species. In particular, species-specific natural mortality rate estimates can be obtained from the following relationship:
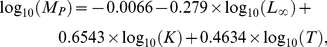
(4)


where 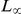 and *K* are VBGF parameters, and *T* is the mean environmental temperature in the population's habitat [26]. As an approximate annual average sea surface temperature for the GBR, we set *T* = 25.8°C for both species [38].

#### Chen and Yuan (*M_CY_*)

Chen and Yuan [39] modified Hoenig's method to estimate natural mortality, by using VBGF parameters to estimate the expected longevity of an unfished population:
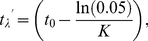
(5)

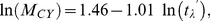
(6)


where Equation 5 is obtained from the VBGF (Equation 1), by setting *t* = *t_λ_*, and solving, assuming, on the basis of an assessment of age-at-length data for fished populations, that 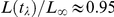 [39].

#### Chen and Watanabe (*M_CW_*)

Chen and Watanabe [40] formulated a three-phase, age-dependent natural mortality schedule. This method uses the assumption that mortality rate is inversely proportional to 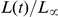 until the end of the reproductive life span, at which point mortality rate increases quadratically with further increases in age:
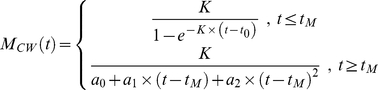
(7a)

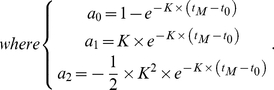
(7b)



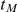
 is age at commencement of senescence. If growth follows the VBGF, and 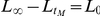 (where 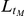 is body length at 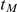 and 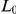 is length at birth), then 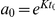. Setting this equal to *a*_0_ in Equation 7b, and solving for 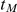, yields:
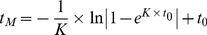
(8)


#### Jensen (*M_JT_* and *M_JK_*)

Natural mortality rate can be derived from relationships commonly termed “Beverton-Holt live history invariants”. Specifically, life history considerations, and analysis of empirical data, indicate that the dimensionless quantities 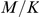 and 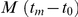 are approximately constant across a broad range of taxa [41]. According to Jensen [28], maturation occurs at approximately the inflection point of the von Bertalanffy growth curve for weight, in which case these two constants are 1.5 and 1.65, respectively. This yields two alternative estimates of mortality rate:
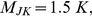
(9)

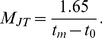
(10)


Note that Jensen [28] derived the latter relationship from the VBGF with *t_0_* set to zero. However, on basis of non-zero size at birth of sharks, we have rederived this relationship more generally, allowing for non-zero size at birth (Equation 10: also see [41]).

### Estimation of Population Growth Rates

In addition to estimating total and natural mortality rate, we determined the long-term population growth rate implied by each of these mortality rates. To do this, we first transformed the mortality rates estimated from each method described above to annual probabilities of survival (*S*(*t*)* = e* ^−*Z(t)*^ or *S*(*t*)* = e* ^−*M(t)*^, where *t* represents age), and then incorporated these estimates into Leslie matrix population models, along with the maturity and fecundity estimates (Text S2). The long-term geometric growth rate of a population is the leading eigenvalue of this matrix.

Use of a Leslie matrix population model requires an estimate of longevity, the maximum age of individuals in the population. Robbins et al. [18] used 19 years, because this was the oldest age of individuals of both species in the catch. However, maximum observed age in a sample can be biased low as an estimate of longevity, particularly for heavily fished populations, because so few individuals survive to reach maximum age. We therefore use a longevity of 25 years as the maximum age in our baseline simulations (this is the maximum reported age globally for these species: [42], [43]). However, we repeated all simulations for both species using a longevity of 19 years, and again using variable longevity based on the bootstrap distribution of longevity obtained in the calculation of *M_CY_* (Equation 5). These two alternative estimates probably exceed the reasonable range of longevity values expected for these species (see *Discussion*).

### Comparison of Estimates

We estimated the differences between pairs of methods used to estimate total mortality rate, to assess whether any of them yielded estimates that were significantly different from each other. We repeated this procedure for our estimates of natural mortality rate. We also wished to obtain “consensus” estimates, which incorporate information from each of the methods applied. Ideally, we would do this by weighting each model according to some assessment of the relative strength of evidence, or subjective prior belief, for it (*sensu* [44], [45]). However, because the models were not parameterized from the same data (e.g., *Z_HF_* uses Hoenig's [27] data on the maximum observed age and mortality rates of fishes, *Z_HC_* uses the cetacean data, *Z_BH_* uses neither, etc), and we know of no general assessments of the relative robustness of these different estimates that could be used to assign prior probabilities, this was not possible. Instead, we calculate a simple average of the estimates obtained from our different methods.

### Characterization of Uncertainty

We applied non-parametric bootstrapping to rigorously characterize uncertainty in our estimates of mortality rates and long-term population growth rates. Specifically, we produced two “best estimates” of mortality rate and population growth rate: those based on the actual (raw) empirical data (i.e., using maximum likelihood parameter estimates without regard to the bootstrap replicates), hereafter termed the “sample estimates,” and median bias-corrected estimates obtained using standard bootstrap bias-correction procedures [46]. We calculated bias-corrected, accelerated (BCa) bootstrap confidence intervals [46], using the jackknife method to account for the fact that the bootstrap distributions combine information from multiple data sets [47]. We also applied the bootstrap method to bias-correct, and characterize the uncertainty in, the consensus estimates of population growth rate, as well as the differences between individual estimates of population growth rate.

A detailed explanation of the bootstrap algorithms is presented in the Supplementary Material (Text S3).

### Projection of Abundance Differences

Finally, we wished to compare differences in abundance between fished and unfished reefs, obtained in previous studies via UVS, with the differences implied by the natural and total population growth rates estimated here. All reefs previously surveyed [18], [32] were originally open to fishing, so we assume all reefs had comparably depleted reef shark densities when protection commenced. If so, the density independent long-run rate of growth of the ratio of fished and unfished population sizes would follow:
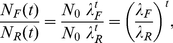
(11)


where *N*_0_ is initial abundance, *N_R_*(*t*) and *N_F_*(*t*) are the abundances in the reserve and fished areas after *t* years, and *λ_R_* and *λ_F_* are the population growth rates in the unfished and fished areas. Note that this approach is only approximate, because it assumes negligible dispersal between fished and unfished populations, and because density-dependent processes should eventually begin limiting population growth on unfished reefs as abundances recover. Nevertheless, to determine whether the estimated population growth rates are on the order required to account for observed differences in abundance on the GBR, we project how abundance differences should develop over time, and we compare these projections with abundance differences estimated from previous visual censuses [18], [32]. For model projections, uncertainty was quantified by bootstrapping as described above. For the visual censuses, the uncertainty in surveyed abundance differences was calculated using Monte-Carlo simulation, assuming a negative binomial distribution of abundances within each population (fished or unfished), with a mean and variance equal to what was observed in the data.

Because both underwater census studies found large, statistically significant differences in abundance between strictly-enforced, no-entry zones, and nominally no-take zones, we used data from no-entry zone reefs to represent “unfished” populations, while open fishing zone reefs were used to represent fished populations. Neither Robbins et al. [18], nor Ayling and Choat [32], found any evidence of significant differences between reefs within management zones, so transects were combined across reefs and treated as the replicates in the Monte Carlo simulations. Because Robbins et al. [18] sampled no-entry reefs only in the northern GBR, and preliminary analysis showed significant differences in *T. obesus* abundance between fished reefs in the northern and the central sectors of the GBR, we only used the northern sector counts from Robbins et al. [18] in our analyses.

## Results

**Figure 2:**
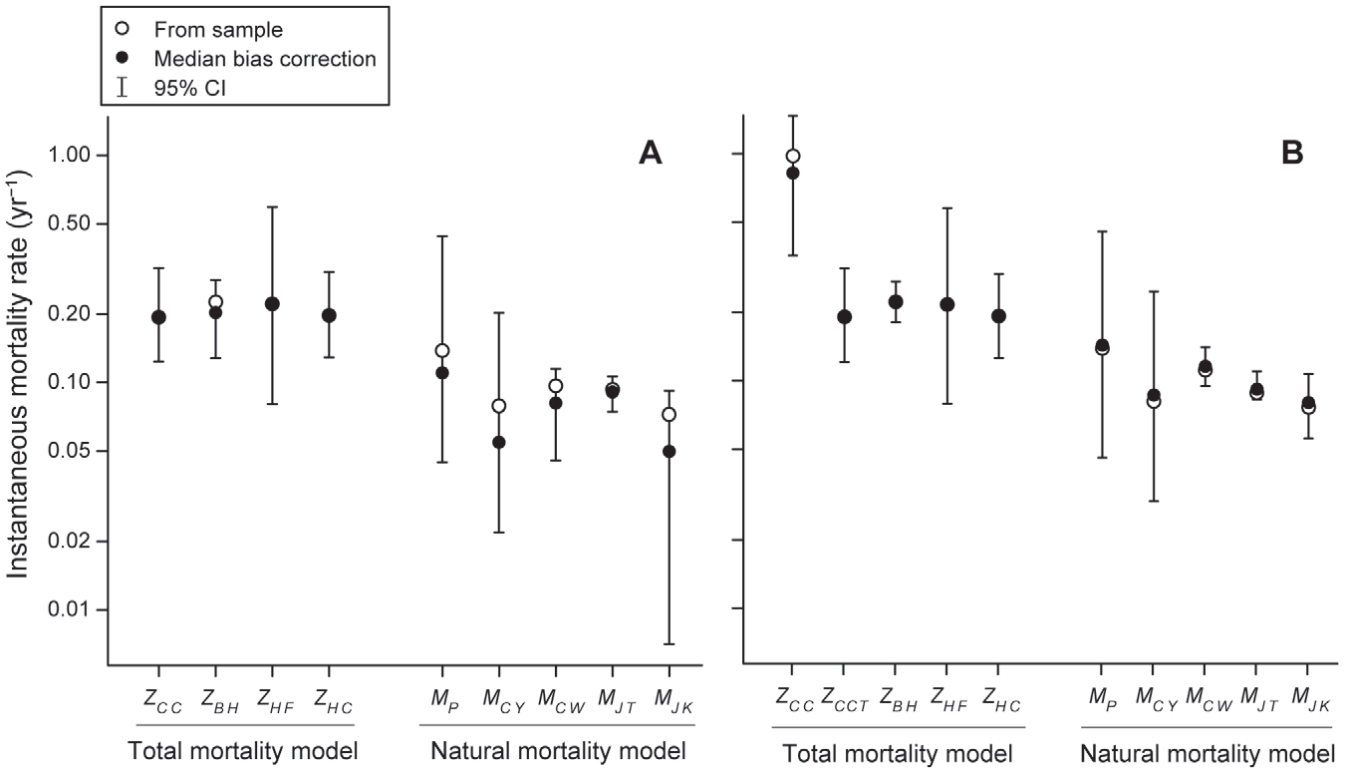
Estimates of natural and total mortality rate for two reef shark species. These estimates were calculated for (A) *T. obesus* and (B) *C. amblyrhynchos* on the Great Barrier Reef. For each method, an open circle indicates the (raw) sample estimate, which was obtained using maximum likelihood parameter estimates from analysis of the empirical data, a closed circle indicates the bootstrap bias-corrected estimate, and whiskers indicate 95% bias-corrected, accelerated (BCa) bootstrap confidence intervals. For methods that produced age-specific estimates of mortality rate (*Z_CC_* for *C. amblyrhynchos*, and *M­_CW_* for both species), an age-averaged mortality rate is shown (i.e., weighted according to the fraction of the population at each age in the stable age distribution). Note that the vertical axis is plotted on log-scale.

**Figure 3:**
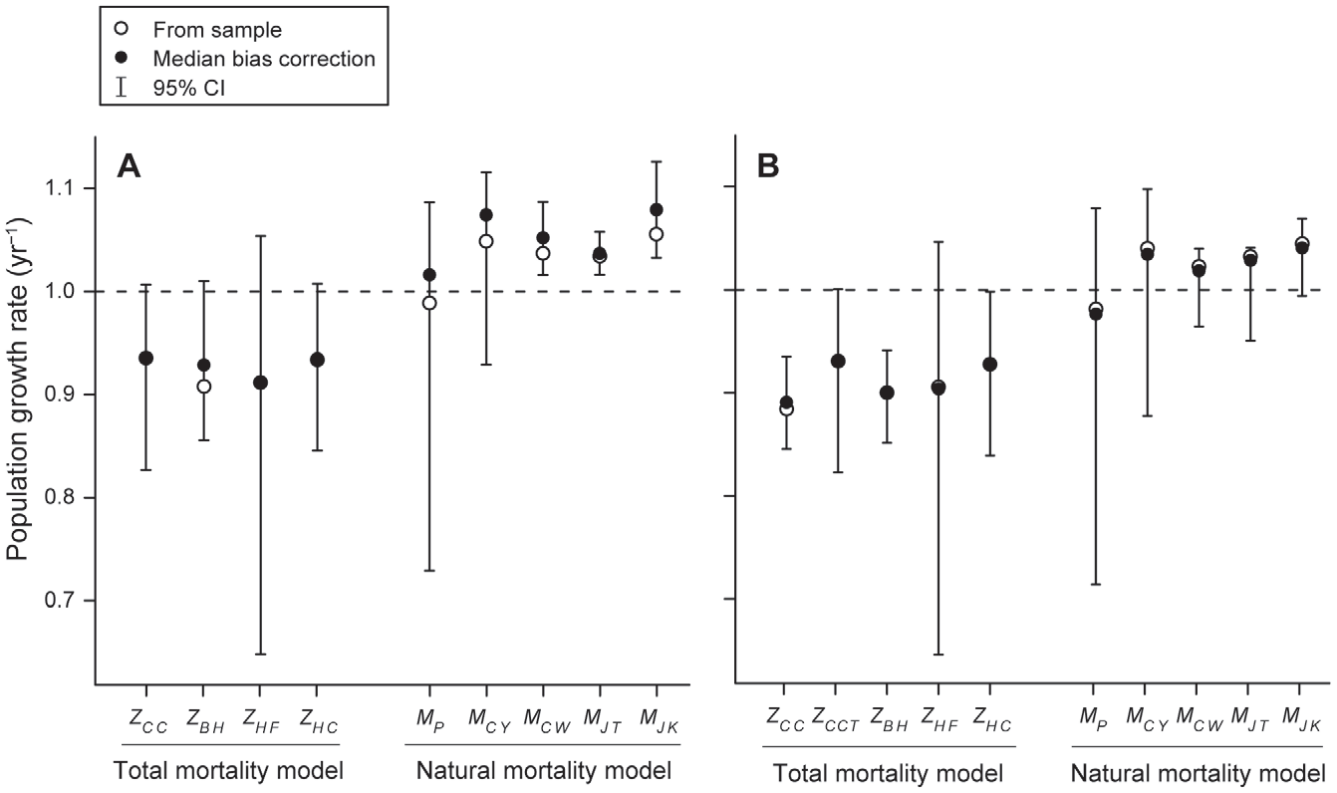
Estimates of natural and total population growth rate for two reef shark species. These estimates were calculated for (A) *T. obesus* and (B) *C. amblyrhynchos* on the Great Barrier Reef. For each method, an open circle indicates the (raw) sample estimate, which was obtained using maximum likelihood parameter estimates from analysis of the empirical data, a closed circle indicates the bootstrap bias-corrected estimate, and whiskers indicate 95% bias-corrected, accelerated (BCa) bootstrap confidence intervals. Dashed lines indicate the threshold between population growth (above) and decline (below).

**Figure 4:**
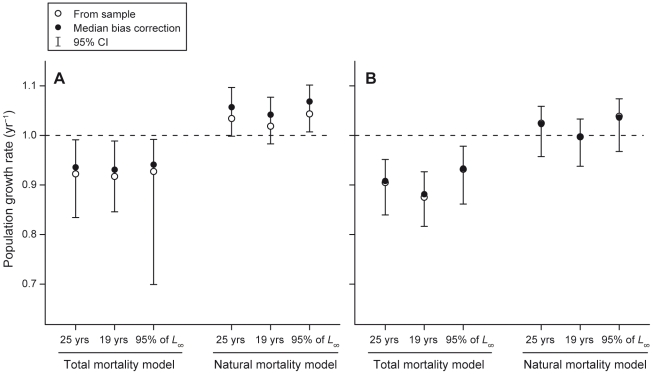
Estimates of consensus natural and total population growth rate for two reef shark species. Estimates were obtained with models with longevity set to 25 years, 19 years and time to achieve 95% of *L_∞_* (sample estimate: 52 and 50 years, respectively) for (A) *T. obesus* and (B) *C. amblyrhynchos* on the Great Barrier Reef. For each method, an open circle indicates the (raw) sample estimate, which was obtained using maximum likelihood parameter estimates from analysis of the empirical data, a closed circle indicates the bootstrap bias-corrected estimate, and whiskers indicate 95% bias-corrected, accelerated (BCa) bootstrap confidence intervals. Dashed lines indicate the threshold between population growth (above) and decline (below).

In general, estimated bias-corrections were small, relative to the estimated sampling variances (evidenced by the breadth of confidence intervals in Figures 2, 3, 4). Because results are correspondingly virtually identical, regardless of whether we bias-correct estimates or not, we discuss only the bias-corrected estimates in the text below.

### Mortality Rates

Natural mortality rate and total mortality rate estimates were internally highly consistent with one another (Figure 2). For total mortality rate, estimates were in the range 0.19–0.23 year^−1^ for both species, except for the catch-curve estimate for *C. amblyrhynchos*, for which age-averaged mortality rate was considerably higher than estimates from other methods. Similarly, natural mortality rate estimates were broadly consistent with one another, ranging from 0.04–0.17 year^−1^. The consistency in natural mortality rate estimates was somewhat surprising, given *M_CY_* yielded estimates of longevity (Equation 5) of both species (52 years for *T. obesus* and 50 years for *C. amblyrhynchos*) more than 2.5 times greater than the maximum observed age in our sample (19 years for both). Conversely, *M_CW_* yielded estimated ages of commencement of the senescence phase (Equation 8) that were much smaller (10 and 14 years for *T. obesus* and *C. amblyrhynchos*, respectively).

### Population Growth Rates

Median bias-corrected estimates of total population growth were highly consistent, ranging from 0.91–0.94 year^−1^ for *T. obesus*, and 0.89–0.93 year^−1^ for *C. amblyrhynchos* (Figure 3). Indeed, when uncertainty was accounted for, none of the estimates differed significantly from one another (Table 1). For natural population growth rate, bias-corrected estimates tended to be lower for *M_P_* than for the life history-based estimates, but, again, after accounting for uncertainty, *M_P_* did not differ significantly from any of the alternatives (Table 1). Nevertheless, for *T. obesus*, *M_JK_* differed significantly from *M_JT_* and *M_CW_*, and for *C. amblyrhynchos*, *M_CW_* differed significantly from *M_JK_* (Table 1). However, this difference was due to the very narrow confidence intervals associated with these three estimates, rather than differences that were large in magnitude (median differences were ∼2% in these cases).

**Table 1:** Pair-wise comparisons of population growth rate estimates (model 2 – model 1) obtained from different mortality models, including 95% bootstrap bias-corrected, accelerated (BCa) confidence intervals.

	Models compared		Difference in best estimates of population growth rate (95% CI^a^)			
Mortality type	Model 1	Model 2	*T. obesus*		*C. amblyrhynchos*	
Total	*Z_CC_*	*Z_CCT_*	NA		−0.04	(−0.12, 0.08)
	*Z_CC_*	*Z­_BH_*	0.00	(−0.01, 0.05)	−0.01	(−0.07, 0.04)
	*Z_CC_*	*Z_HF_*	0.03	(−0.14, 0.34)	−0.01	(−0.16, 0.30)
	*Z_CC_*	*Z_HC_*	0.00	(−0.12, 0.13)	−0.04	(−0.12, 0.08)
	*Z_CCT_*	*Z_BH_*	NA		0.03	(−0.08, 0.12)
	*Z_CCT_*	*Z_HF_*	NA		0.03	(−0.14, 0.34)
	*Z_CCT_*	*Z_HC_*	NA		0.01	(−0.12, 0.13)
	*Z_BH_*	*Z_HF_*	0.03	(−0.25, 0.45)	0.00	(−0.15, 0.29)
	*Z_BH_*	*Z_HC_*	0.00	(−0.17, 0.14)	−0.03	(−0.10, 0.08)
	*Z_HF_*	*Z_HC_*	−0.02	(−0.33, 0.14)	−0.02	(−0.34, 0.13)
Natural	*M_P_*	*M_CY_*	−0.06	(−0.50, 0.09)	−0.06	(−0.33, 0.09)
	*M_P_*	*M_CW_*	−0.04	(−0.39, 0.05)	−0.04	(−0.29, 0.05)
	*M_P_*	*M_JT_*	−0.02	(−0.31, 0.05)	−0.05	(−0.30, 0.04)
	*M_P_*	*M_JK_*	−0.06	(−0.45, 0.03)	−0.06	(−0.31, 0.03)
	*M_CY_*	*M_CW_*	0.02	(−0.16, 0.07)	0.02	(−0.12, 0.07)
	*M_CY_*	*M_JT_*	0.04	(−0.10, 0.07)	0.01	(−0.13, 0.06)
	*M_CY_*	*M_JK_*	0.00	(−0.20, 0.06)	−0.01	(−0.14, 0.05)
	*M_CW_*	*M_JT_*	0.01	(0.00, 0.02)	−0.01	(−0.02, 0.00)
	*M_CW_*	*M_JK_*	−0.03	(−0.04, −0.01)^b^	−0.02	(−0.03, −0.01)^b^
	*M_JT_*	*M_JK_*	−0.04	(−0.06, −0.01)^b^	−0.01	(−0.03, 0.01)

a Confidence intervals were obtained from comparisons of 10 000 bootstrap estimates on the bootstrap-by-bootstrap basis so that the uncertainty distribution accounted for statistical covariances of model parameters (see Text S3 for details).

b Denotes that the two models yielded significantly different estimates of population growth rate (i.e., 95% confidence intervals did not encompass zero).

Consensus estimates of total population growth indicated strong support for the conclusion that both populations are in decline, with at least 95% confidence (Figure 4). For *T. obesus*, the median bias-corrected consensus estimate was 0.94, and for *C. amblyrhynchos*, it was 0.91. Although decreasing longevity to 19 years, or using the bootstrap distribution of *t_λ_* (Equation 5), which implies much greater longevity (50+ years), decreased and increased these population growth rates, respectively, the effects were relatively small: median bias-corrected total population growth rate, and 95% confidence limits, changed by only 1% for *T. obesus*, and 2–3% for *C. amblyrhynchos* (Figure 4).

Consensus estimates of natural population growth indicated the potential for population growth in the absence of fishing: median bias-corrected estimates were 1.06 for *T. obesus*, and 1.02 for *C. amblyrhynchos*, although 95% confidence limits on the population growth rate of *C. amblyrhynchos* did include values below replacement (Figure 4). Again, using alternative longevity measures did not change estimated natural population growth rates, or their 95% confidence limits, by more than ∼2%.

### Projection of Abundance Differences

**Figure 5:**
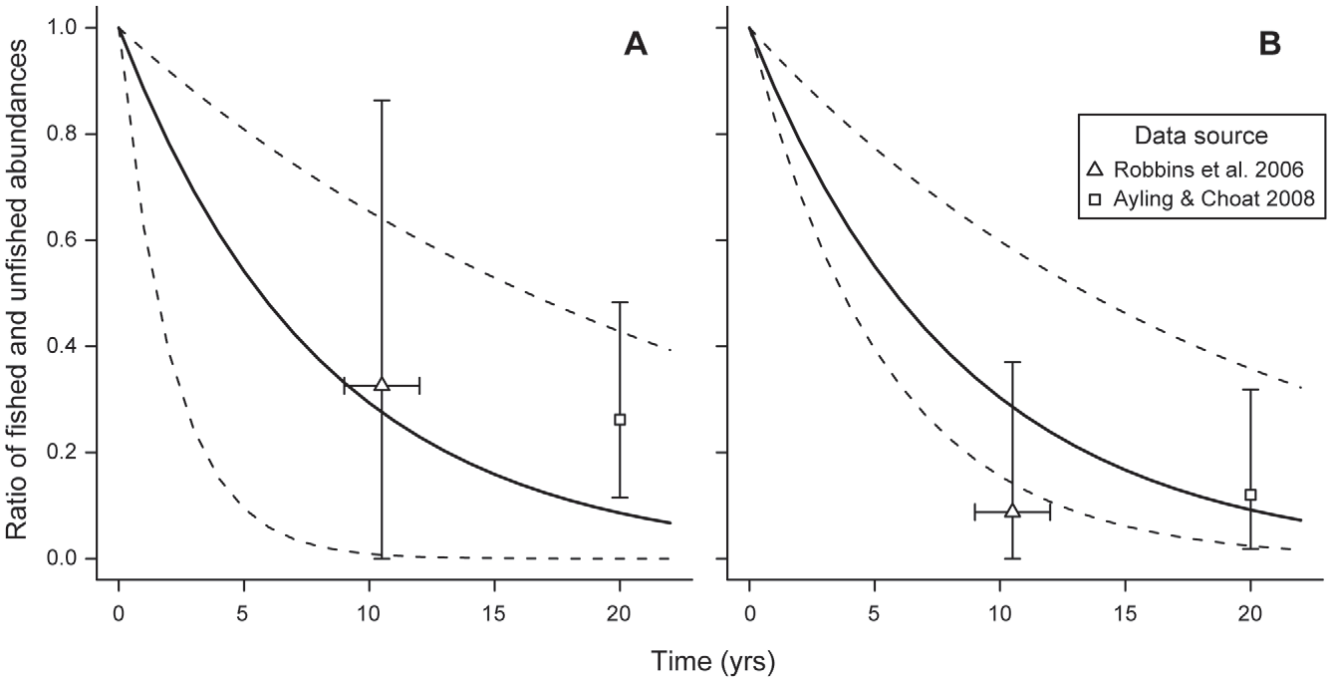
Estimates of ratio of fished to unfished abundances over time for two reef shark species. These estimates were calculated for (A) *T. obesus* and (B) *C. amblyrhynchos* on the Great Barrier Reef, from our bootstrap bias-corrected consensus estimates of long-term total and natural population growth rates (Equation 11). The dashed lines indicate 95% bias-corrected, accelerated (BCa) bootstrap confidence intervals on these abundance ratios. The mean differences between surveyed abundances on open fishing and unfished (no-entry) reefs are plotted as triangles and squares for the data in Robbins et al. [18] and Ayling and Choat [31], respectively. Location of triangles and squares on the horizontal axis reflects the number of years elapsed between establishment of the relevant no-entry zones, and the underwater surveys. For Robbins et al. [18], horizontal whiskers reflect the fact that surveys took place over a multi-year period (2001–2004). Vertical whiskers indicate 95% bootstrap confidence intervals on these abundance ratios, generated by Monte-Carlo simulation, as described in the *Methods*.

Consensus estimates of natural and total population growth rate imply a per-annum density-independent long-run rate of change in the ratio of abundances in fished and unfished populations of 0.88 for *T. obesus*, and 0.89 for *C. amblyrhynchos*. When used to project abundance differences over time, these rates yielded estimates of the ratio of abundances on fished versus unfished reefs that were consistent with previously obtained UVS data (Figure 5), although the *C. amblyrhynchos* data of Robbins et al. [18] suggest potentially greater abundance differences than our model projections (Figure 5B).

## Discussion

Ongoing, rapid declines in many shark species worldwide, coupled with evidence that shark depletion can have substantial, cascading effects on community structure and dynamics, have made assessing the status and trends of shark populations a high priority in conservation biology [5]. This study shows how a robust assessment of mortality and population growth rates can be made using indirect estimates of mortality rate, and can thereby provide essential information for the sustainable management of sharks, and potentially other exploited species for which direct estimates of mortality are unavailable. More specifically, it highlights the vulnerability of reef sharks to overfishing, even in well-managed reef systems like the GBR. Our use of multiple models to estimate rates of population decline, and potential natural population growth, reveals a high degree of concordance between several indirect methods of estimating these two quantities. For total population growth, none of the model estimates differed significantly from one another. For natural population growth, model estimates either did not differ significantly, or differences were very small in magnitude. Our results were also surprisingly insensitive to longevity: even pessimistic (19 years) and very optimistic (50+ years) estimates changed consensus estimates of population growth rate only by 1–3%. The range of longevities explored here almost certainly exceeds the reasonable range. Clearly, since 19-year old sharks were observed in the catch data, longevity cannot be less than this. Conversely, the longevity of our optimistic scenario (50+ years) is highly unlikely, given that no shark older than 19 years appeared in the 333 sharks in the combined commercial and research catches (including line and spear fishing), and that no shark of either species older than 25 years has been reported anywhere in the world [42]. In any case, the conclusion that substantial population declines in *T. obesus* and *C. amblyrhynchos* have been occurring, largely as a consequence of increased mortality from fishing, appears highly robust to the available indirect methods of estimating total mortality rate for these species.

None of our estimates of total population growth rate differed significantly from one another, despite differences in assumptions associated with our different estimates of total mortality rate. Catch curve analysis assumes the catch composition is representative of the survivorship of the underlying population (i.e., if the catch curve indicates 30% fewer individuals at one age than the next-younger age, this indicates that survival between these ages is 70%). This assumption requires that there are no consistent temporal trends in numbers recruiting to the fishery, or post-recruitment age differences in catchability. A trend of declining recruitment, or greater susceptibility of older individuals to fishing, for instance, both tend to make fitted catch curves shallow, relative to the underlying survivorship pattern, and thus under-estimate mortality. The Beverton-Holt method is likely to be similarly biased by trends in recruitment or age-dependent post-recruitment catchability, because mean age in the catch (inversely related to *Z_BH_*) would be biased high in the same cases where *Z_CC_* would be biased low. In particular, it is possible that the high abundance of very young *C. amblyrhynchos* in the catch indicates over-representation (due, for instance, to naiveté or a tendency for older sharks to spend less time in shallow water, where fishing activity is greater). Such an effect would bias *Z_CC_* high. However, there is no evidence of such over-representation in the *T. obesus* catch data; moreover, this species is less aggressive towards bait than *C. amblyrhynchos* [18], [35]. Thus, if anything, we would expect *Z_CCT_* (use of the *T. obesus* catch curve as an estimate of *C. amblyrhynchos* mortality rate) to be biased low. The fact that our estimates of population growth rate for *C. amblyrhynchos* did not differ significantly, regardless of which catch curve we used, suggests that any associated biases are probably small, relative to the uncertainties. Moreover, Hoenig's method, which yields estimates that do not differ from *Z_BH_*, *Z_CC_*, or *Z_CCT_*, is based on empirical relationships that hold across species, and does not require any assumptions about the representativeness of the catch data for our study species. Hoenig's method should only be biased if our study species are outliers, relative to the species used in the construction of the regression model. We have minimized the risk of this by applying both relationships calibrated for fish (*Z_HF_*) and cetaceans (*Z_HC_*); moreover, the two elasmobranch species used in the construction of the *Z_HF_* regression model are not outliers [27].

For natural mortality, the only assumption shared by all methods is that growth is well-characterized by the von Bertalanffy function: VBGF growth parameters are covariates in the regression model used to calculate *M_P_*, and putative life-history relationships involving VBGF parameters are involved in the derivation of the other estimates. However, this assumption is well-met for these species (Text S1). The derivation of *M_CY_*, *M_JT_*, and *M_JK_* all require an assumption of constant mortality rate, but *M_CW_* explicitly incorporates age-dependent mortality rates. Moreover, *M_P_*, like Hoenig's method, is empirical and not derived from any life-history assumptions. For estimates of natural population growth rate obtained from these methods, we found significant differences involving only Jensen's estimates. We suspect that these estimates, which had very narrow confidence limits, underestimate the true uncertainty associated with the use of proposed life-history invariants to estimate demographic rates. In particular, as the term implies, application of this method assumes that, for all species, natural mortality rate follows Equation 9 or Equation 10 exactly, and thus the only uncertainty associated with this estimate is the uncertainty associated with the estimation of the von Bertalanffy growth parameters *K* and *t*_0_, and age at maturity *t_m_*. However, there is an emerging consensus that these quantities are not truly invariant, but instead represent a central tendency or average across species, with individual species deviating somewhat from these average relationships [48]. The magnitude of this interspecific variation has not yet, to our knowledge, been estimated, but it implies that the uncertainties around *M_JK_* and *M_JT_* (and, by implication, the consensus estimate of natural population growth) are likely to be underestimated to some extent.

The consensus estimates of natural and total population growth imply population growth rates consistent with other lines of evidence. For instance, our projections of abundance differences are consistent with UVS estimates on the GBR (Figure 5). In addition, Smith et al. [37] estimated natural population growth rates of approximately 4–6% per year for similarly sized sharks (including *T. obesus* and *C. amblyrhynchos*), which is in good agreement with our consensus estimates of natural population growth rate (Figure 4). However, the lack of a marked decline in GBR catch rates has been used to suggest that rapid declines in abundances of these two species are unlikely [33]. As noted earlier, we believe that commercial landings are unlikely to provide reliable estimates of catch rates, due to the tendency for killed and discarded sharks to be under-reported [15]. Moreover, changes in factors that influence catch, such as targeting behavior or fishing gear, can cause catch rates to stabilize even when populations are declining, a phenomenon known as hyperstability [49]. Estimates of catch rates from boats with scientific observers on board, such as the Effects of Line Fishing (ELF) experiment [33], are likely to be more reliable. Nevertheless, Heupel et al. [33] noted that accurate recording of these catch rates for sharks was only emphasized from about 2000, five years before completion of the study. Given the short duration of the resulting time series, it is possible that a comprehensive accounting of uncertainty in potential population trends, focusing on this five-year period, may well yield results that are more consistent with our model projections, and earlier visual abundance surveys, than these authors initially supposed.

Accounting for uncertainty when making management decisions, and ensuring that the information on which such decisions are made are robust to the simplifying assumptions of particular models, are important objectives when using science to inform policy making. Demonstrating that such considerations have been taken into account has become increasingly imperative in many policy contexts, where criticism of the handling of uncertainty, or of the simplifying assumptions associated with particular models, is used to challenge the legitimacy of inferences drawn from models, even where contrary evidence is absent [50]. In the marine environment, demographic data are often sparse and, in some cases, direct information about some demographic parameters may be lacking, ambiguous, or subject to potentially large biases, particularly for mortality rate. Such problems are likely to be particularly common for populations that have low commercial value, are caught as bycatch, or are managed by countries with limited resources for fisheries data collection or analysis. Although multiple indirect estimates of mortality have been used in other studies (e.g., [51]–[53]), only one study has quantified the uncertainty associated with these estimates [54], and none have quantitatively examined the consistency of estimates or integrated them with the uncertainty associated with other demographic rates in an assessment of population status. By doing so here for two reef shark species, we have shown that several of these methods provide surprisingly consistent estimates of natural and total mortality rate, and of the population growth rates that they imply, once the uncertainty associated with the individual estimates is taken into account. Moreover, simple consensus estimates of natural population growth are broadly consistent with other lines of evidence, such as abundance differences on fished and unfished reefs, and estimates of “rebound potential” for similar-sized shark species [18], [32], [37]. For the GBR at least, this concordance of evidence appears to justify management actions to substantially reduce the fishing mortality of reef sharks. More broadly, we believe that our study demonstrates that this approach may be applied to a broad range of exploited species for which direct estimates of mortality are ambiguous or lacking, leading to improved estimates of population growth.

## Supporting Information

Text S1 **Demographic data and parameter estimation.**
(DOC)

Text S2 **Population model framework.**
(DOC)

Text S3 **Bootstrap algorithms for characterization of uncertainty.**
(DOC)
