# Supplementary material for: Population Growth Rates of Reef Sharks with and without Fishing on the Great Barrier Reef: Robust Estimation with Multiple Models
Source: PLoS One. 2011 Sep 23;6(9):e25028. doi: 10.1371/journal.pone.0025028 (PMC3179482; doi:10.1371/journal.pone.0025028)
Supplement: Text S1 — Demographic data and parameter estimation. (DOC) [file pone.0025028.s001.doc]

# **Equation**Supplementary Text **S1**

# Summary of demographic data and parameter estimation

**Catch, Maturity and Fecundity**

Catch, maturity, and fecundity data are described in detail elsewhere [1], so what follows is a summary. Catch data are comprised of 134 *T. obesus* and 199 *C. amblyrhynchos* collected at multiple reefs at both northern (14°25’S-14°42’S) and central (18°02’S-19°14’S) Great Barrier Reef (GBR), Australia. The samples were obtained from both commercial and field collections using spear- and line-fishing between 2001 and 2005. All sharks were aged by counting growth bands in sectioned vertebrae, and assuming annual band-pair deposition.

We based the analysis of maturity on sexual maturity state (0 for immature, 1 for mature) data recorded from a total of 67 *T. obesus* and 75 *C. amblyrhynchos* females. We estimated probability of maturity as a function of age with a logistic regression (as per [1]). We employed age at which 50% of the age-class reaches maturity as *t_mat_* (e.g. [2]) in the calculation of *M_JT_*. The best estimate of age at 50% maturity from the regression was 8 yrs for *T. obesus* with a 95% bootstrap confidence interval of 8-9 yrs, and 11 yrs for *C. amblyrhynchos* with a 95% bootstrap confidence interval of 10-12 yrs.

We estimated average litter sizes from the number of embryos present in 14 and 9 pregnant females in the sample of *T. obesus* and *C. amblyrhynchos*, respectively. The observed litter sizes exhibited no apparent change with respect to age for *T. obesus* and were simply averaged for this species. Mean litter size was 2.1 by the raw data with a 95% bootstrap confidence interval of 1.7-2.5. On the other hand, the data for *C. amblyrhynchos* contained greater numbers of embryos in older females. Robbins et al. [1] found that a sigmoid function provided good fit the data:

$N_{pup}\left(t\right)=1+\frac{a-1}{1+10^{b\times\left(c-t\right)}}$,

where *t* = age (in years), *N_pup_* = the number of pups per pregnant female, and *a*, *b* and *c* are fitted parameters. *a* is an upper asymptote and given *a* > 1, 1 is a lower asymptote. *b* is a measure of the steepness of increase towards *a*, and *c* is the inflection point (in years) of the curve. Estimates of these parameters from the best-fitted curve to the raw data were *a*=3.5, *b*=1.1, and *c*=13.4. For both species, we halved these estimates of average litter sizes to account for 1:1 sex ratio of gestating pups, and halved further for the biennial periodicity of breeding.

**Growth**

We fitted the three-parameter von Bertalanffy growth curve to age-length data from 125 *T. obesus* and 89 *C. amblyrhynchos* by ordinary least squares regression. Comparisons by Akaike Information Criterion (AIC) favored the use of different growth curves for females and males of *T. obesus*, but not of *C. amblyrhynchos*. (Table S1.1). Therefore, for all subsequent analyses, we combined the data from both sexes for *C. amblyrhynchos*, but we used the data of females only for *T. obesus*. Plots of residuals against age confirm good-fit of the von Bertalanffy growth model to the data, with no evidence of bias or skew (Figure S1.1). The 95% bootstrap confidence intervals of growth parameters were *L_∞_* = (167, 236), *K* = (0.03, 0.09), and *t_0_* = (-12.2, -6.9) for *T. obesus* and *L_∞_* = (205, 279), *K* = (0.03, 0.07), and *t_0_* = (-9.8, -6.5) for *C. amblyrhynchos*.

**Table S1.1.** Best estimates of von Bertalanffy growth parameters and goodness-of-fit statistics by Akaike Information Criterion for a comparison of sex-combined and sex-specific model fits.

|  | Data | $L_{\infty}$(cm) | $K$(yr^-1^) | $t_{0}$(yr) | Residual SD | AIC |
| --- | --- | --- | --- | --- | --- | --- |
| *T. obesus* | | | | | | |
|  | Female | 208 | 0.05 | -9.8 | 6.16 | 790.5 |
|  | Male | 153 | 0.10 | -6.8 | 4.95 |  |
|  | Sex-combined | 202 | 0.05 | -9.8 | 5.81 | 797.6 |
| *C. amblyrhynchos* | | | | | | |
|  | Female | 219 | 0.06 | -7.2 | 6.09 | 577.3 |
|  | Male | 241 | 0.05 | -7.7 | 6.41 |  |
|  | Sex-combined | 229 | 0.05 | -7.5 | 6.21 | 571.7 |


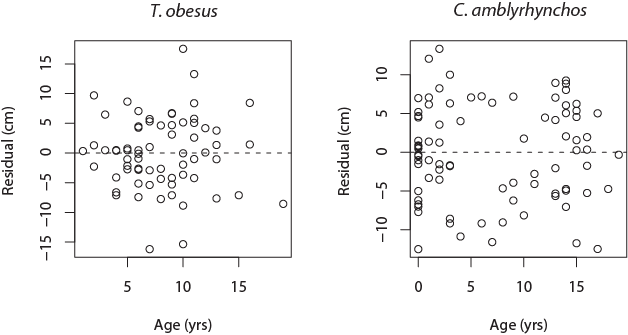


**Figure S1.1.** Residuals of observed lengths from the best-fit of the von Bertalanffy growth model plotted against age for *T. obesus* and *C. amblyrhynchos* on the Great Barrier Reef.

**References:**

1. Robbins WD, Hisano M, Connolly SR, Choat JH (2006) Ongoing collapse of coral-reef shark populations. Current Biology 16: 2314-2319.
2. Beverton RJH (1992) Patterns of reproductive strategy parameters in some marine teleosts fishes. Journal of Fish Biology 41(Supplement B): 137-160.
