# Supplementary material for: Population Growth Rates of Reef Sharks with and without Fishing on the Great Barrier Reef: Robust Estimation with Multiple Models
Source: PLoS One. 2011 Sep 23;6(9):e25028. doi: 10.1371/journal.pone.0025028 (PMC3179482; doi:10.1371/journal.pone.0025028)
Supplement: Text S2 — Population model framework. (DOC) [file pone.0025028.s002.doc]

# Supplementary Text S2

# Summary of population model framework

**Leslie Matrix Population Model**

We utilized an age-based matrix population model which contains age-specific annual survival probabilities and fecundities as in Robbins et al. [1]:

$A=\left[\begin{matrix} \begin{matrix} 0 \\ s_{0} \\ 0 \\ 0 \\ 0 \end{matrix} & \begin{matrix} \begin{matrix} F_{1} \\ 0 \\ s_{1} \\ 0 \\ 0 \end{matrix} & \begin{matrix} \begin{matrix} \cdots\\ \\ \ddots\\ \\ \cdots\end{matrix} & \begin{matrix} \begin{matrix} F_{n-1} \\ 0 \\ 0 \\ 0 \\ s_{n-1} \end{matrix} & \begin{matrix} F_{n} \\ 0 \\ 0 \\ 0 \\ 0 \end{matrix} \end{matrix} \end{matrix} \end{matrix} \end{matrix}\right]$,

where *s_t_* is per-capita annual survival probability for age *t* (years) and *F_t_* is per-capita fertility for age *t* and *n* is the longevity. This matrix was constructed for each of the methods we applied for mortality estimates. *F_t_* are products of probability of maturity at age *t*-1(to account for the ~1 year gestation period: [2]), and the mean litter size at age *t*.

**References:**

1. Robbins WD, Hisano M, Connolly SR, Choat JH (2006) Ongoing collapse of coral-reef shark populations. Current Biology 16: 2314-2319.
2. Uchida S, Toda M, Kamei Y (1990) Reproduction of elasmobranchs in captivity. In: Pratt HL Jr, Gruber SH, Taniochi T, editors. NOAA technical report NMFS 90. Elasmobranchs as living resources: advances in the biology, ecology, systematic, and the status of the fisheries.. Seattle: United States Department of Commerce. pp. 211-237.
