# Supplementary material for: Population Growth Rates of Reef Sharks with and without Fishing on the Great Barrier Reef: Robust Estimation with Multiple Models
Source: PLoS One. 2011 Sep 23;6(9):e25028. doi: 10.1371/journal.pone.0025028 (PMC3179482; doi:10.1371/journal.pone.0025028)
Supplement: Text S3 — Bootstrap algorithms for characterization of uncertainty. (DOC) [file pone.0025028.s003.doc]

# Supplementary Text S3

# Bootstrap algorithms applied for characterization of uncertainty

The parameter estimates of our Leslie matrix model came from four different data sets (catch data, fecundity data, maturity data, and growth data), and these data sets were frequently used to estimate multiple model parameters. For this reason, there are statistical covariances between some of the model parameters. If not appropriately accounted for, these covariances may lead to excessively wide or narrow confidence intervals, depending on whether the covariances are positive or negative, Therefore, to implicitly incorporate covariances in model parameters, we used a single bootstrap replicate of each data set to estimate all of the parameters required for each bootstrap replicate Leslie matrix. For instance, a logistic regression on the maturity data is required to calculate all of the fecundity parameters in the model, and it is also used to calculate Jensen’s maturity-based natural mortality rate (*M_JT_*). Therefore, for any given bootstrap replicate population growth rate estimate, the same bootstrap maturity data set was used to estimate each of these parameters. Similarly, to account for any statistical covariances between different estimates of mortality rate (e.g., a bootstrap replicate of catch data may cause both catch curve analysis and the Beverton-Holt method to yield unusually low or high mortality rates), each bootstrap replicate of a difference between population growth rates, and of a consensus population growth rate, used the same bootstrap sample for each of the alternative mortality rate estimates. This procedure was then repeated 10 000 times to yield bootstrap distributions for differences between population growth rate estimates, and consensus estimates of population growth rate.

For estimation of VBGF growth parameters, older individuals were scarce in our dataset, but these points were important for reliable estimation of *L_∞_*. Therefore, we employed weighted bootstrapping so that each age class had an equal probability to appear in a bootstrap replicate [1].

For *Z_HF_, Z_HC_, M_P_,* and *M_CY_*, we also incorporated prediction error associated with the regressions, by randomly sampling from a normal distribution with standard deviation equal to the residual standard error of the fitted regression models [2,3]. This residual standard error represents the unexplained variation among species in the relationship between mortality rate and the explanatory variables in the respective regression models.

**References:**

1. Efron B, Tibshirani RJ (1998) An introduction to the bootstrap. Boca Raton: Chapman and Hall. 436 p.
2. Hoenig JM (1983) Empirical use of longevity data to estimate mortality rates. Fishery Bulletin 82: 898-903.
3. Pauly D (1980) On the interrelationships between natural mortality, growth parameters, and mean environmental temperature in 175 fish stocks. ICES Journal of Marine Science 39: 175-192.
